# Astrocyte hepcidin is a key factor in LPS-induced neuronal apoptosis

**DOI:** 10.1038/cddis.2017.93

**Published:** 2017-03-16

**Authors:** Lin-Hao You, Cai-Zhen Yan, Bing-Jie Zheng, Yun-Zhe Ci, Shi-Yang Chang, Peng Yu, Guo-Fen Gao, Hai-Yan Li, Tian-Yu Dong, Yan-Zhong Chang

**Affiliations:** 1 Laboratory of Molecular Iron Metabolism, The Key Laboratory of Animal Physiology, Biochemistry and Molecular Biology of Hebei Province, College of Life Science, Hebei Normal University, Shijiazhuang, China; 2 School of Basic Medical Sciences, Hebei Medical University, Shijiazhuang, China

## Abstract

Inflammatory responses involving microglia and astrocytes contribute to the pathogenesis of neurodegenerative diseases (NDs). In addition, inflammation is tightly linked to iron metabolism dysregulation. However, it is not clear whether the brain inflammation-induced iron metabolism dysregulation contributes to the NDs pathogenesis. Herein, we demonstrate that the expression of the systemic iron regulatory hormone, hepcidin, is induced by lipopolysaccharide (LPS) through the IL-6/STAT3 pathway in the cortex and hippocampus. In this paradigm, activated glial cells are the source of IL-6, which was essential in the iron overload-activated apoptosis of neurons. Disrupting astrocyte hepcidin expression prevented the apoptosis of neurons, which were able to maintain levels of FPN1 adequate to avoid iron accumulation. Together, our data are consistent with a model whereby inflammation initiates an intercellular signaling cascade in which activated microglia, through IL-6 signaling, stimulate astrocytes to release hepcidin which, in turn, signals to neurons, via hepcidin, to prevent their iron release. Such a pathway is relevant to NDs in that it links inflammation, microglia and astrocytes to neuronal damage.

Neurodegenerative diseases (NDs) comprise a multitude of progressive degenerative diseases of the central nervous system (CNS), including Alzheimer's disease (AD), Parkinson's disease (PD), amyotrophic lateral sclerosis and multiple sclerosis. Although oxidative stress, mitochondrial dysfunction, excitotoxicity and apoptotic processes have been identified as being involved in neuronal degeneration,^1, 2^ the etiology and pathogenesis of neurodegeneration have not yet been fully elucidated. Accumulating evidence indicates that iron metabolism dysregulation is a key factor in the pathogenesis of NDs.^3, 4, 5^

Iron is an essential trace element in various physiological processes, such as oxygen transport (hemoglobin), redox reactions, neurotransmitter synthesis, myelin production, energy metabolism and other mitochondrial functions.^6, 7, 8^ However, due to its propensity to release electrons and produce reactive oxygen species (ROS), excess iron can result in oxidative stress and cause cellular damage. Iron chelation has been shown to provide neuroprotection, preventing apoptosis and activating cellular protection pathways against oxidative stresses.^9, 10^ Thus, iron levels must be tightly controlled in order to prevent cellular damage in brain, while maintaining sufficient iron for essential cellular functions.

Hepcidin is a peptide hormone known as the key systemic regulator of iron homeostasis. Hepcidin acts as a negative regulator of cellular iron release, by binding to ferroportin 1 (FPN1), this only known iron exporter, causing its internalization and degradation.^11^ Although hepcidin is predominantly expressed in the liver, it is also detectable in different brain areas, including the olfactory bulb, cortex, hippocampus, amygdala, thalamus, hypothalamus, mesencephalon, cerebellum pons, spinal cord and dorsal root ganglia.^12, 13, 14^ Lateral cerebral ventricle (LCV) injection of hepcidin has been shown to decrease FPN1 levels and result in brain iron overload in the cerebral cortex, hippocampus and striatum.^13^ The same study demonstrated decreases in FPN1 levels and a concomitant blockage of iron release in neurons following hepcidin treatment. The localization and functional activity of hepcidin in the brain implicates hepcidin in an important role in maintaining brain iron homeostasis.

Much evidence is available to indicate that inflammation contributes to the development of NDs. Furthermore, intravenous injection of pro-inflammatory lipopolysaccharide (LPS) can regulate the expression of hepcidin not only in peripheral organs but also in the brain.^15^ Previous studies have also shown that hepcidin is expressed in both microglia and astrocytes, and hepcidin levels are considerably increased by inflammatory stimuli *in vitro*.^16^ Inflammation can lead to hepcidin production through the direct activation of Toll-like receptors (TLRs) or through inflammatory cytokines such as interleukin-6 (IL-6).^17, 18, 19^ IL-6 signaling results in the phosphorylation of signal transducer activator of transcription 3 (STAT3) which, in turn, promotes the transcription of the gene encoding hepcidin.^20^ These previous studies thus suggest that hepcidin is not only a central factor in the regulation of iron metabolism but also a critical linker between iron homeostasis and the modulation of acute inflammatory responses.^21^

Microglia are immune cells resident in the parenchyma of the CNS that play an essential role in the maintenance of the brain microenvironment. They constantly survey the extracellular milieu and rapidly initiate inflammatory responses to infection or injury.^22^ The role of astrocytes, other innate immune cells in the CNS, in immune and inflammatory responses is garnering increasing attention. Astrocytes also sense infection and injury, and amplify immune reactions initiated by microglia.^23, 24, 25^ In fact, both microglia and astrocyte activation are required for effective immune responses in the brain. However, in recent years, increasing evidence strongly suggests that sustained inflammatory responses involving activated microglia and astrocytes contribute to the pathogenesis of NDs.^26^ Despite the connection between inflammation and iron metabolism, the relationship, and mechanisms involved, between neuroinflammation and iron metabolism in NDs is not clear.

In the current study, we found that iron accumulation contributes to neuronal apoptosis induced by LPS. During this process, activated astrocytes and microglia play a critical role in neuronal iron accumulation; LPS-induced hepcidin expression in astrocytes is regulated by IL-6 from activating microglia. Astrocyte-derived hepcidin was tightly correlated with the apoptosis of neurons through iron accumulation in the neurons. These results indicate that astrocyte hepcidin links neuroinflammation to a dysregulation of iron metabolism and may therefore further elaborate the pathogenesis of NDs.

## Results

### High levels of oxidative stress play an important role in apoptosis induced by LPS

**Figure 1:**
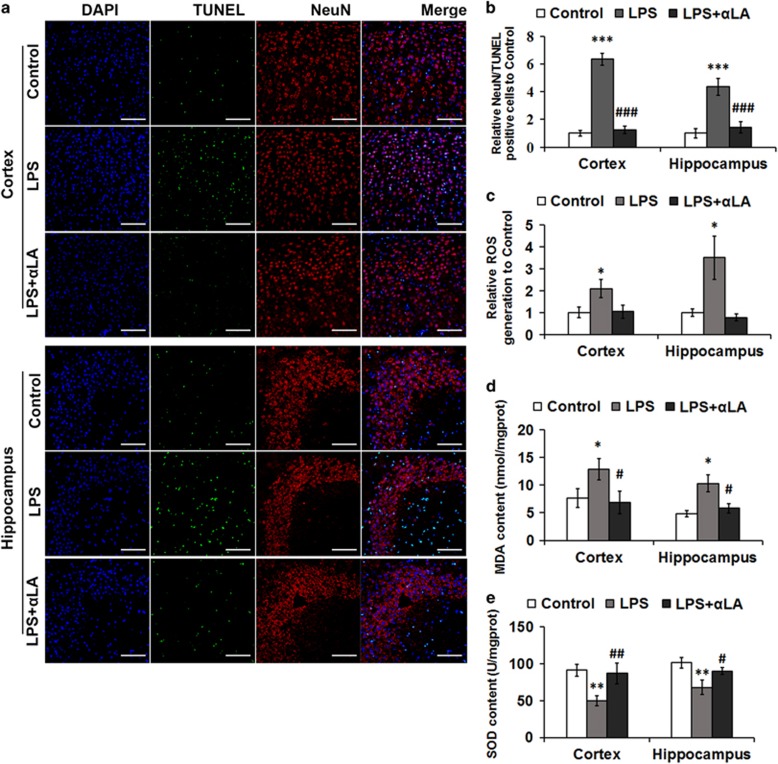
LPS induces apoptosis and oxidative stress in the cortex and hippocampus. (**a**) Apoptosis was assayed by DAPI, NeuN and TUNEL staining. Scale bar=100 *μ*m. (**b**) The number of NeuN/TUNEL-positive cells were counted in five separate fields and are presented as a percentage of the total cells in the fields. Values are presented as the mean±S.D. ***P<0.001 *versus* control group; ^###^*P*<0.001 *versus* LPS treatment group. *n*=6. (**c**–**e**) Analysis of ROS, MDA and SOD levels in the cortex and hippocampus following LPS and *α*LA treatment. Values are presented as the mean±S.D. *P<0.05, **P<0.01 *versus* control group; ^#^*P*<0.05, ^##^*P*<0.01 *versus* LPS group. *n*=6

The NeuN/TUNEL assay was utilized to elucidate the effect of neuroinflammation induced by LPS on neurons in the cortex and hippocampus. The number of NeuN/TUNEL-positive cells in both regions (Figures 1a and b) was significantly increased following intracerebroventricular (ICV) LPS injection.

Oxidative stress is one of several factors that can cause neuronal injury. ROS damage membranes, proteins and nucleic acids, ultimately leading to both apoptosis and necrosis. To study whether increased levels of oxidative stress are responsible for the LPS-induced apoptosis observed in the cortex and hippocampus, we examined the levels of ROS, superoxide dismutase (SOD) and malondialdehyde (MDA) after LPS treatment. Compared to the control groups, the levels of ROS and MDA (Figures 1c and d) increased significantly, while SOD levels (Figure 1e) declined markedly in the LPS groups in the two brain regions.

Alpha-lipoic acid (*α*LA) is a compound with strong antioxidant properties. It readily passes across the blood brain barrier and can provide considerable defense against tissue damage induced by free radicals. We examined the number of NeuN/TUNEL-positive cells and the levels of ROS, MDA and SOD after *α*LA and LPS treatment and found that *α*LA treatment can significantly reduce the number of NeuN/TUNEL-positive cells (Figures 1a and b) and rescue the changes in ROS, MDA and SOD levels (Figures 1c–e) in LPS-treated mice. This indicates that a high level of oxidative stress likely contributes to the activation of neuron apoptosis after LPS treatment.

### Excessive iron accumulation in neurons, caused by downregulated FPN1, contributes to the elevated oxidative stress induced by LPS

**Figure 2:**
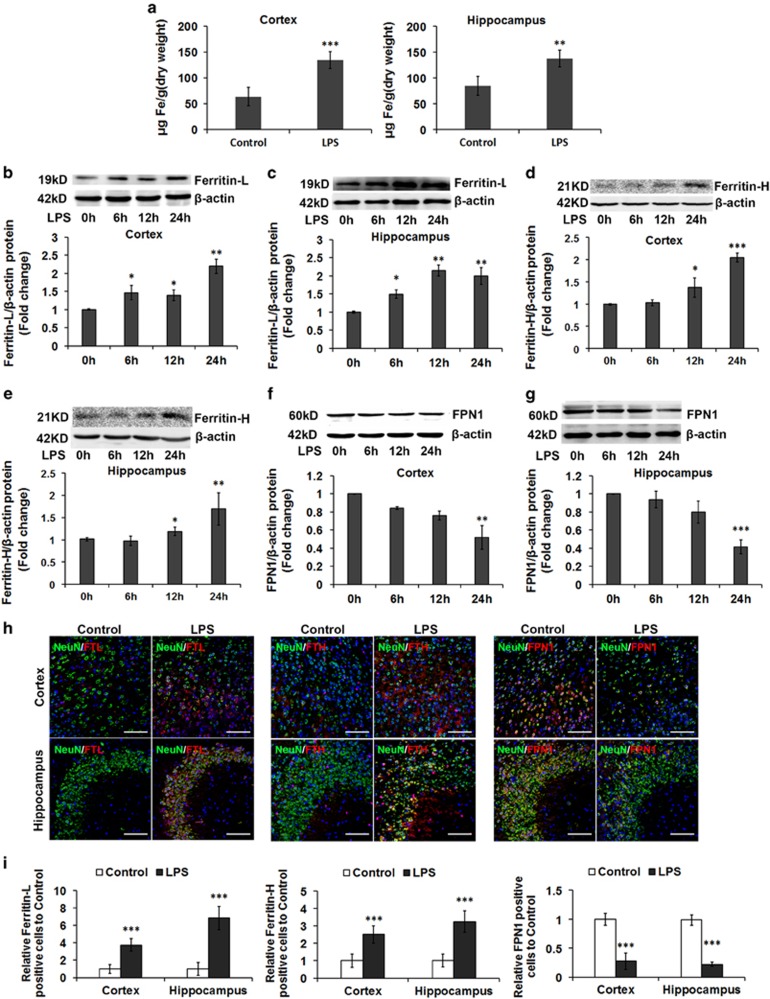
LPS induces iron accumulation in the cortex and hippocampus. (**a**) ICP-MS analysis of total iron levels in the cortex and hippocampus following LPS treatment. Values are presented as the mean±S.D. **P<0.01, ***P<0.001 *versus* control group. *n*=6. (**b**–**g**) The protein levels of Ferritin-L, Ferritin-H and FPN1 in these regions of brain at 0, 3, 6, 12 and 24 h after LPS injection. Expression levels were normalized to *β*-actin and presented as the mean±S.D. *P<0.05, **P<0.01 and ***P<0.001 *versus* 0 h. *n*=6. (**h** and **i**) Double immunofluorescence labeling of Ferritin-L, Ferritin-H or FPN1 (red) and NeuN (green, staining for neurons) was carried out in cortex and hippocampal sections 24 h after LPS treatment. DAPI was used for nuclear staining. Scale bar=100 *μ*m. Values are presented as the mean±S.D. ***P<0.001 *versus* control group. *n*=6

Excessive iron accumulation can generate ROS and exacerbate oxidative stress through Fenton chemistry. To investigate the causes of the high level of oxidative stress, we examined the iron levels in the cortex and hippocampus following LPS injection directly, by inductively coupled plasma mass spectroscopy (ICP-MS), and indirectly, by western blot analysis of iron-related proteins. We found that the total iron levels (Figure 2a) were markedly upregulated in these two brain regions in the LPS-treated groups, compared with control groups. In addition, LPS-treated mice exhibited a significant increase in Ferritin-L, a protein involved in the storage of excess iron, in the two brain regions (Figures 2b and c). The increase in Ferritin-L levels in the cortex and hippocampus at 24 h after LPS treatment is about twice that in untreated animals (Figures 2b and c). Similarly, the LPS-treated mice also exhibited a significant increase in Ferritin-H in the two brain regions at 24 h after LPS treatment (Figures 2d and e). To further investigate the neuronal iron levels, we used immunofluorescence (IF) staining to simultaneously detect NeuN-positive neurons and the levels of Ferritin-L or -H protein expression. The double IF staining results show that both isoforms were significantly increased in neurons at 24 h after LPS treatment in both regions (Figures 2h and i).

To explore the mechanisms of LPS-induced iron accumulation, we quantified FPN1 protein levels in the cortex and hippocampus after LPS administration at 0, 6, 12 and 24 h, respectively. We found that FPN1 expression decreased in a time-dependent manner in the examined brain regions, reaching the lowest point at 24 h after LPS administration (Figures 2f and g). The double IF staining results show a significant decrease in FPN1-positive neurons at 24 h after LPS treatment in both regions (Figures 2h and i).

The total amount of intracellular iron is a balance of its release and uptake.^27^ With this in mind, we also assessed the iron uptake-related proteins in LPS-treated mouse brains. In particular, we examined transferrin receptor 1 (TfR1)^27^ and divalent metal transporter 1 (DMT1).^28, 29^ We found that TfR1 and DMT1 (IRE) protein amounts either declined or were negligibly altered after LPS administration (Supplementary Figure S1). The double IF staining results show that TfR1 and DMT1 followed the same trend as above in neurons at 24 h after LPS treatment in both regions (Supplementary Figure S2). Thus, the iron accumulation in neurons induced by LPS is achieved by a reduction of FPN-mediated export.

### LPS increases the expression of hepcidin, through the IL-6/STAT3 axis

**Figure 3:**
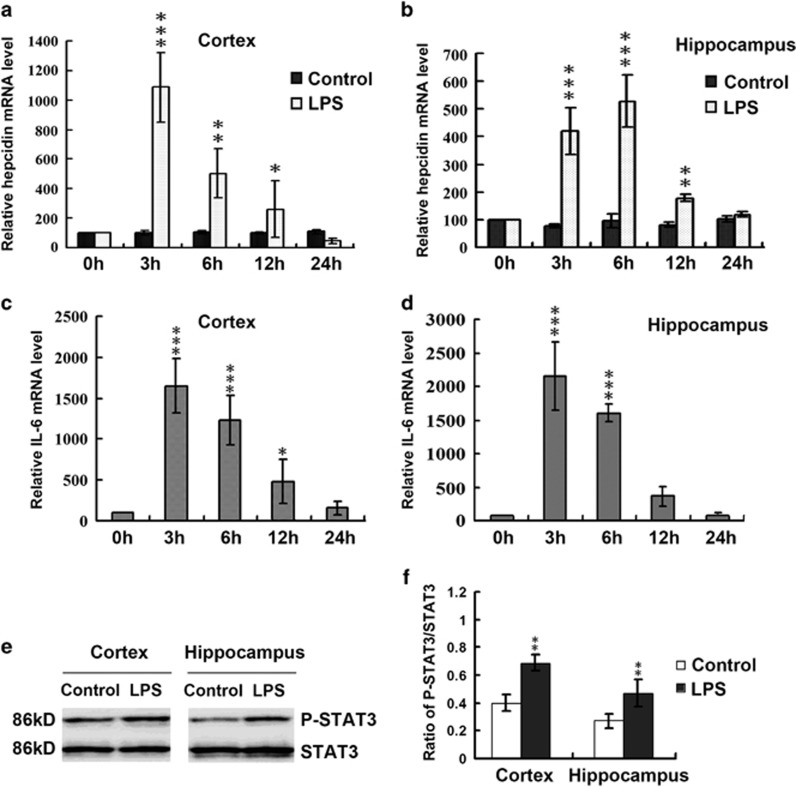
LPS induces increased expression of hepcidin through upregulating the IL-6/STAT3 pathway *in vivo*. Hepcidin (**a** and **b**) and IL-6 (**c** and **d**) mRNA expression in the cortex and hippocampus at 0, 3, 6, 12 and 24 h after LPS injection in the lateral cerebral ventricle. Expression levels were normalized to *β*-actin and presented as the mean±S.D. *P<0.05, **P<0.01 and ***P<0.001 *versus* 0 h. *n*=6. (**e** and **f**) STAT3 phosphorylation levels at 3 h following LPS injection in the cortex and hippocampus. The ratio of P-STAT3 and STAT3 are presented as the mean±S.D. **P<0.01 *versus* control group. *n*=6

Previous work has demonstrated that hepcidin downregulates iron efflux via the internalization and degradation of FPN1.^11^ Some groups^30^ have also demonstrated hepcidin upregulation, and consequent FPN1 decreases, following LPS treatment in the brain. In our study, LPS treatment led to a significant induction of hepcidin mRNA expression in these brain tissues, while saline-treated controls exhibited no significant changes in hepcidin mRNA expression. In the LPS group, hepcidin expression markedly increased until a peak at 3 h in the cortex and 6 h in the hippocampus. After this time, though decreasing, the hepcidin expression remained above controls through the 12 h time point in these regions (Figures 3a and b).

Among the different stimuli that induce hepcidin expression, cytokines (tumor necrosis factor-*α* (TNF-*α*), IL-6), erythropoietic activity, iron stores and hypoxia are the most common.^31, 32^ Interestingly, IL-6 is induced within 3 h after LPS injection. Urinary hepcidin has also been shown to increase after LPS treatment. The latter peaked within 6 h in 10 healthy individuals, which is congruent with an active IL-6-hepcidin axis in the context of hypoferremia in inflammation.^33^ IL-6 directly regulates hepcidin through the induction and subsequent promoter binding of signal transducer and activator of transcription 3 (STAT3).^20^ To explore the molecular mechanisms involved in LPS-induced hepcidin expression, we performed additional experiments to measure the levels of IL-6 mRNA and STAT3 phosphorylation (P-STAT3) after LPS administration into the brain. IL-6 mRNA maintained a significantly high level at 3 and 6 h and decreased at 24 h (Figures 3c and d). The P-STAT3/STAT3 ratio was markedly increased at 6 h after LPS administration (Figures 3e and f).

### LPS-induced hepcidin expression in astrocytes is regulated by microglia-derived IL-6

**Figure 4:**
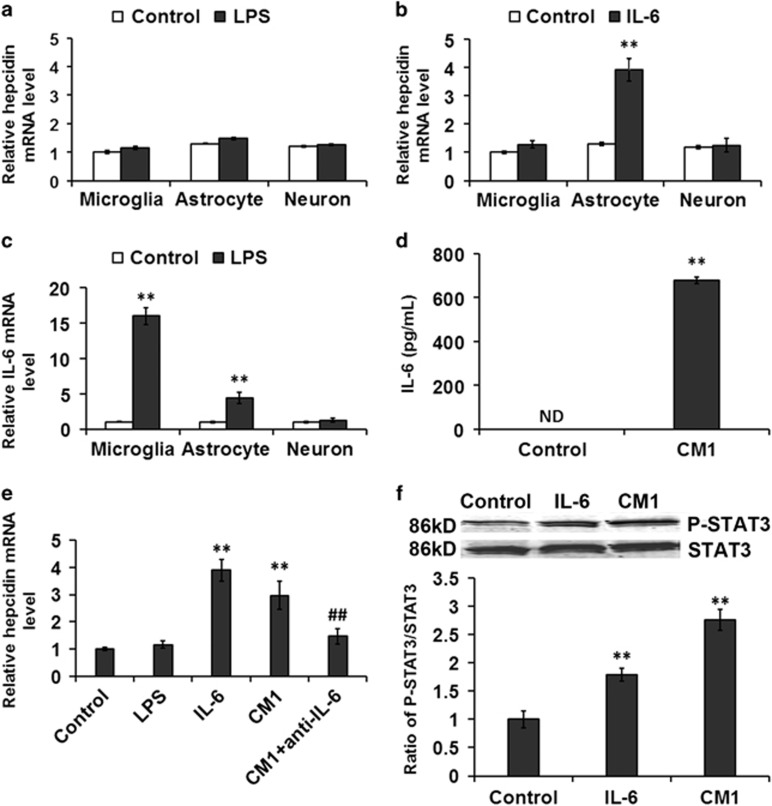
LPS-induced hepcidin expression in astrocytes is regulated by IL-6 from activated microglia. (**a**) Hepcidin mRNA expression in microglia, astrocytes and neurons following LPS treatment for 6 h. (**b**) Hepcidin mRNA expression in microglia, astrocytes and neurons following IL-6 treatment for 6 h. Data are presented as the mean±S.D. **P<0.01 *versus* control group. *n*=6. (**c**) IL-6 mRNA expression in microglia, astrocyte and neuron following LPS treatment for 6 h. Data are presented as the mean±S.D. **P<0.01 *versus* control group. *n*=6. (**d**) The levels of IL-6 release were assayed with ELISA in CM1 following by LPS treatment. CM1: microglia medium collected 24 h after LPS stimulation. ND, non-detectable. **P<0.01 *versus* control group. *n*=6. (**e**) Hepcidin mRNA expression in astrocyte at 6 h following LPS, IL-6, CM1 and CM1+anti-IL-6 treatment. CM1+anti-IL-6: add IL-6 antibody to CM1. Data are presented as the mean±S.D. **P<0.01 *versus* control group; ^##^*P*<0.01 *versus* CM1 treatment group. *n*=6. (**f**) STAT3 phosphorylation levels at 6 h following IL-6 and CM1 treatment. The ratio of P-STAT3 and STAT3 is presented as the mean±S.D. **P<0.01 *versus* control group. *n*=6

To determine the cells responsible for LPS-mediated hepcidin upregulation, we evaluated the responses of primary rat microglia, astrocytes and neurons to LPS. Interestingly, the hepcidin mRNA levels showed no significant change in cultures of pure microglia, astrocytes or neurons 24 h after LPS treatment (Figure 4a). However, hepcidin mRNA was upregulated in astrocytes in response to IL-6 treatment for 24 h, but not in microglia or neurons (Figure 4b).

To further determine the mechanism of LPS-induced hepcidin expression, we examined the respective IL-6 expression and secretion in the three types of cells. IL-6 mRNA was induced approximately 20-fold in microglia, 4-fold in astrocytes and no significant change in primary neurons at 24 h of LPS treatment (Figure 4c). Meanwhile, we detected about 700 pg/ml IL-6 in microglia-conditioned medium collected 24 h after LPS stimulation (CM1) (Figure 4d).

To further determine whether hepcidin mRNA levels are indirectly induced by LPS via the generation and release of IL-6 from microglia, we measured the hepcidin mRNA levels in astrocytes exposed to CM1 with or without a neutralizing IL-6 antibody. As predicted, CM1 was able to induce hepcidin expression; however, IL-6 antibody abrogated the increased expression of hepcidin induced by the treatment (Figure 4e). In addition, the ratio of P-STAT3/STAT3 increased dramatically in astrocytes after IL-6 or CM1 treatment (Figure 4f). Together, these results indicate that microglia are the initial responders to LPS-mediated inflammation, activating and releasing IL-6, which, in turn, stimulates astrocytes to express hepcidin.

### Astrocytes hepcidin production leads to neuronal iron accumulation and tightly correlates with LPS-mediated apoptosis

**Figure 5:**
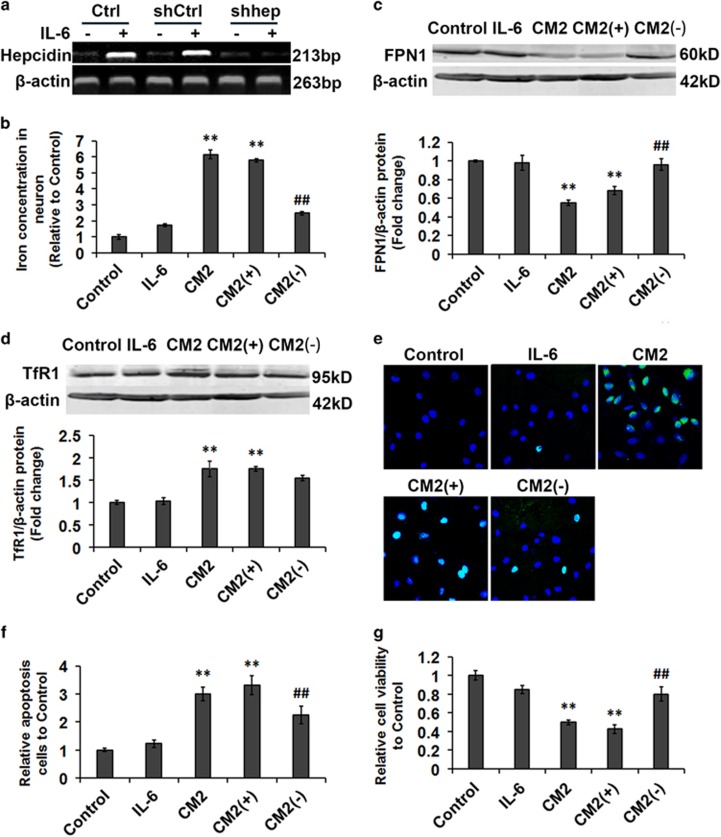
Reduced astrocyte hepcidin inhibits LPS-induced apoptosis of neurons by permitting sufficient expression of FPN1 to prevent toxic iron accumulation. (**a**) Hepcidin mRNA expression in C6 cell infected with shCtrl- or shHepcidin-lentivirus, following IL-6 treatment. (**b**) Total iron of primary neurons was measured by ICP-MS after IL-6, CM2, CM2 (+) and CM2 (−) treatment for 24 h. All data represent the mean value of five separate experiments (three replicates per experiment). Data are presented as the mean±S.D. **P<0.01 *versus* control group; ^##^*P*<0.01 *versus* CM2 (+). *n*=6. (**c** and **d**) FPN1 and TfR1 protein levels were detected by western blotting lysates from primary neurons after IL-6, CM2, CM2 (+) or CM2 (−) treatment for 6 h. Relative expression levels were normalized to *β*-actin and presented as the mean±S.D. **P<0.01 *versus* control group; ^##^*P*<0.01 *versus* CM2 (−) treatment group. *n*=6. (**e**) Primary neurons were stained with DAPI and TUNEL, then examined by fluorescence microscopy following IL-6, CM2, CM2 (+) and CM2 (−) treatment for 24 h. (**f**) Statistical analysis of apoptosis of primary neurons using TUNEL staining. Data are presented as the mean±S.D. **P<0.01 *versus* control group; ^##^*P*<0.01 *versus* CM2 (+) treatment group. *n*=6. (**g**) Statistical analysis of cell viability as detected by MTT after IL-6, CM2, CM2 (+) and CM2 (−) treatment for 24 h. Data are presented as the mean±S.D. **P<0.01 *versus* control group; ^##^*P*<0.01 *versus* CM2 (+) treatment group. *n*=6. CM2: conditioned medium from C6 cells 24 h after IL-6 treatment. CM2 (+): conditioned medium from C6 cells which were infected with shHepcidin-lentivirus, collected 24 h after IL-6 treatment. CM2 (−): conditioned medium from C6 cells which were infected with shCtrl-lentivirus, collected 24 h after IL-6 treatment

Since astrocytes appear to be the major hepcidin-expressing cell type in the brain, we tested the effects of hepcidin produced by astrocytes on the iron metabolism of neurons. IL-6 treatment exceedingly induced the transcription of hepcidin in both the control group (C6 cells) and the shCtrl group (C6 cells transfected with the empty vector), but not in the shHep group (C6 cells transfected with lentiviral shhepcidin). Astrocyte C6 cells were infected with shHepcidin-lentivirus to disrupt the expression of hepcidin following IL-6 treatment (Figure 5a). We will refer to the C6 cell-conditioned medium collected 24 h after IL-6 treatment as ‘CM2', that of the cells transfected with a control shRNA as ‘CM2 (+)' and that of cells transfected with the shHepcidin as ‘CM2 (−)'. We evaluated the iron concentration in neurons by ICP-MS following IL-6, CM2, CM2 (−) or CM2 (+) treatment. Iron accumulated dramatically in neurons treated with CM2 or CM2 (+), when compared with the CM2 (−) group. The iron concentration of neurons in the IL-6 treatment group showed no significant change compared with control (Figure 5b). Our results suggest that blocking hepcidin release from astrocytes can significantly inhibit the neuronal iron accumulation observed following LPS treatment.

To investigate the mechanism responsible for iron accumulation in cultured neurons treated with the conditioned media, the protein levels of FPN1 and TfR1 were detected following CM treatment. Consistent with the iron accumulation data, the expression of FPN1 was significantly decreased in neurons after CM2 or CM2 (+) treatment, while the medium conditioned by cells with blocked hepcidin had a significantly diminished effect (Figure 5c). The expression of TfR1 was not significantly altered in either the CM2 (+) or the CM2 (−) treatment group (Figure 5d). These results indicate that the stimulated expression of hepcidin in astrocytes may downregulate the expression of FPN1 in neurons leading to their iron accumulation.

To determine whether astrocyte hepcidin plays a part in LPS-mediated neuronal apoptosis, we assayed apoptosis and neuronal survival rate in primary rat neurons treated with CM. No significant apoptosis was observed (DAPI/TUNEL staining) following IL-6 treatment, in contrast to the increased apoptosis produced by CM2 or CM2 (+) treatment. Compared with the CM2 treatment group, the apoptosis rate of neurons was significantly reduced in the CM2 (−) treatment group (Figures 5e and f). Next, we evaluated neuronal survival, as determined by MTT assay, following CM treatment. No significant neuronal death was observed following IL-6 treatment, whereas cell viability decreased when CM2 or CM2 (+) was used. The cell survival rate of neurons was only slightly decreased in the CM2 (−) treatment group, compared with controls (Figure 5g). In addition, we assessed apoptosis, cell viability and iron concentration in neurons induced by LPS, CM1 and CM3. Of these, only CM3 treatment was able to increase the number of apoptotic neurons, reduce cell viability and elevate iron concentration (Supplementary Figure S3). CM3 was astrocyte medium collected 24 h after CM1 treatment (Supplementary Figure S3). Our data are suggestive of a tight correlation between hepcidin release from astrocytes and neuronal apoptosis.

### LPS-mediated astrocyte hepcidin release and subsequent neuronal apoptosis is blocked in mice with conditional knockdown of hepcidin in astrocytes

To further detect the role of astrocyte-released hepcidin in the LPS-mediated apoptosis of neurons, we utilized GFAP-shhepcidin mice, which possess a preferential, astrocyte-specific knockdown of hepcidin. The genotype of the mice used in this study was first verified by primer-specific PCR. Supplementary Figure S4A shows the PCR products resolved by agarose gel electrophoresis. Transgenic mice possess the 457 bp-length PCR product, which was present in all of the mice used in the experiments. Hepcidin expression was also evaluated by PCR in primary cultures of astrocytes extracted from the GFAP-shhepcidin mice. The expression of hepcidin in astrocytes was remarkably decreased (by approximately 60% Supplementary Figure S4B), indicating the high-efficiency of hepcidin knockdown in astrocytes in GFAP-shhepcidin mice.

**Figure 6:**
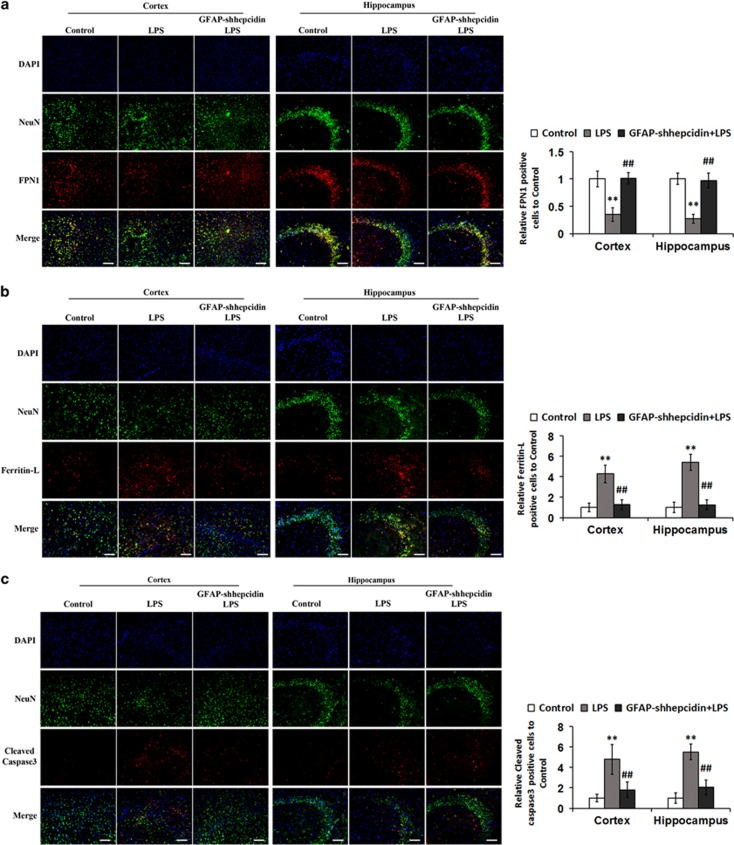
The iron levels and apoptosis in neurons in the cortex and hippocampus in wild-type and GFAP-shhepcidin mice following LPS injection. Double immunofluorescence labeling of FPN1 (**a**), Ferritin-L (**b**) or cleaved caspase 3 (**c**) and NeuN (staining for neurons) was carried out in cortical and hippocampal sections. Scale bar=100 *μ*m. Values are presented as the mean±S.D. **P<0.01 *versus* control group; ^##^*P*<0.01 *versus* LPS group. *n*=6

Next, we assessed the amount of apoptosis and levels of iron in cortical and hippocampal neurons in these mice. A significant decrease in FPN1 protein levels and increase in Ferritin-L levels were observed in neurons after LPS treatment of wild-type mice brains. In contrast, an increased expression of FPN1 (Figure 6a) and decreased expression of Ferritin-L (Figure 6b) in neurons were observed in the GFAP-shhepcidin mice after LPS injection. These mice also exhibited increased protein levels of cleaved caspase 3, a key effector of apoptosis. The expression of caspase 3 in neurons was significantly decreased in GFAP-shhepcidin mice (Figure 6c). These findings suggest that blockage of astrocyte hepcidin generation can protect the neurons from inflammation-stimulated apoptosis by reducing neuronal iron concentration.

## Discussion

Neuroinflammation occurs in association with the pathological changes in the brain of patients who, for example, have undergone brain surgery, have AD or suffer from an ischemic stroke.^34, 35^ Meanwhile, excess iron is considered an important factor contributing to neurotoxicity in several neurodegenerative disorders, including AD.^36^ In addition, inflammation is tightly linked to iron metabolism dysregulation.^16^ However, the clinical consequences of this finding and the dynamics of iron accumulation in AD are widely unknown. Here, our data showed that neuroinflammation induced hepcidin expression in astrocytes, and then induces the apoptosis of neurons through iron accumulation.

At autopsy, the brain of a typical AD patient reveals a gross cerebral atrophy involving brain regions implicated in learning and memory processes, including the temporal, parietal and frontal cortex as well as the hippocampus and amygdala, in part explaining the nature of the clinical AD symptoms. This brain volume reduction is due to a profound degeneration of neurons and synapses.^37^ MRI results showed that AD patients have increased iron levels in the putamen, pulvinar thalamus, red nucleus, hippocampus and temporal cortex.^38^ In addition, neuroinflammation also has been reported as a contributor to the neuropathogenesis of cognitive impairment.^39, 40^ Therefore, we focused on the apoptosis of cortical and hippocampal neurons. Our results suggest that neuroinflammation induced by intracerebral LPS indeed contributes to neuronal apoptosis *in vivo* (Figures 1a and b). Afterward, the oxidative stress level of these brain regions was induced following LPS treatment, through detecting ROS, MAD and SOD levels (Figures 1c–e). To further establish the causal relationship between oxidative stress and cell death, *α*LA, an antioxidant, was used to reduce the level of oxidative stress and to further decrease the apoptosis of neurons following LPS treatment (Figure 1).

Our evidence also suggests that aberrant iron accumulation in the brain may play a pivotal role in the pathogenesis of many diseases involving cognitive dysfunction.^41, 42, 43^ Iron overload is known to cause free radical formation, oxidative stress and neuronal damage.^41, 44, 45, 46^ Neuroinflammation has been found in many iron-associated NDs such as AD.^47^ It has also been shown that iron chelation possess a neuroprotective effect against LPS-induced neuroinflammation and cognitive deficits by decreasing brain iron levels.^40^ Pro-inflammatory cytokines such as IL-1beta activate the translation of H- and L-ferritin, in addition to iron.^48^ A positive correlation between ferritin levels and iron levels is well established.^49, 50^ In our study, iron accumulated was directly examined by ICP-MS and indirectly examined by the analysis of ferritin in the cortex and hippocampus, following ICV injection of LPS *in vivo* (Figure 2). In addition, the relative abundance of H- and L-ferritin depends on the cell type.^51^ Neurons express mostly H-ferritin, microglia express mostly L-ferritin and oligodendrocytes express similar amounts of both subunits.^52, 53^ Therefore, the expression of both ferritin isoforms was detected following LPS treatment. LPS increased the expression of both ferritin isoforms (Ferritin-H and Ferritin-L) in neurons (Figure 2). Based on our data as well as the evidence from the literature, it is highly likely that iron accumulated in the neurons.

To detect the molecular mechanism responsible for this finding, we examined iron content as well as the levels proteins involved in cellular iron metabolism. Our results show that the protein level of the iron exporter, FPN1 (Figures 2f–i), decreased dramatically in neurons *in vivo*, while the iron uptake-related proteins expression slightly decreased or did not change in neurons (Supplementary Figures S1 and S2) after LPS administration in the examined brain regions, suggesting that the iron accumulation was caused by the reduction of iron release, rather than increased iron absorption. Hepcidin is a central iron regulatory hormone. Hepcidin binds to ferroportin 1 (Fpn1) and induces its internalization and degradation.^11^ Therefore, we examined the level of hepcidin in the cortex and hippocampus, after ICV injection of LPS. The mRNA level of hepcidin increased significantly in both the cortex and hippocampus (Figure 3). In addition, the temporal and spatial expression pattern of IL-6 and P-STAT3/STAT3, both known to stimulate hepcidin production, implicated these signals in brain hepcidin elevation (Figure 3).

Increasing evidence has strongly suggested that sustained inflammatory responses involving activated microglia and astrocytes contribute to the pathogenesis of NDs; the inflammatory program that is induced by these cells has the potential to cause neuronal dysfunction and contribute to the severity of several NDs if inflammatory responses are not properly resolved.^26, 54^ Microglia sense infection and injury through numerous pattern recognition receptors, such as TLRs, and secrete inflammatory cytokines, such as TNF-a and IL-1*β*, that direct function in surrounding astrocytes and neurons.^23^ Astrocytes also sense infection and injury, and amplify the immune reaction initiated by microglia.^23–25^ Here, we found that LPS cannot directly induce hepcidin expression in astrocytes, microglia or neurons, but cell culture media conditioned by microglia induced hepcidin release by astrocytes. The hepcidin expression was only induced in astrocytes in response to CM1 or IL-6 treatment (Figures 4a and b). The presence of IL-6 in CM1 and that hepcidin expression was inhibited when IL-6-neutralizing antibodies were added to CM1, indicate that LPS-stimulated microglial IL-6 mediates astrocyte hepcidin production.

Here, we also demonstrate that astrocyte hepcidin, and the downstream neuronal iron accumulation, closely correlate with LPS-mediated apoptosis of neurons. Several lines of evidence suggest that this is due to hepcidin's function as a factor linking iron homeostasis and neuroinflammation. First, we detected the apoptosis of neurons induced by LPS, CM1 and CM3. We utilized this model system in which the apoptosis of neurons was induced by LPS, which is not effectively sensed by neurons and does not directly cause neuronal death. The number of neurons apoptosis increased significantly was induced by CM3 which is astrocyte medium collected 24 h after CM1 treatment (Supplementary Figure S4). Second, the production of IL-6 secreted by microglia did not in itself lead to the apoptosis of neurons. The number of neurons apoptosis only increased significantly was induced by astrocyte medium collected 24 h after IL-6 treatment (Figure 5). Finally, blockage of hepcidin expression in astrocytes resulted in decreased iron overload in neurons and apoptosis in response to LPS both *in vitro* and *in vivo* (Figures 5 and 6).

Despite the large contribution of hepcidin in our model, it should be recognized that other pathways also participate in the inflammatory response, including cytokine-mediated affects elicited by TNF-*α* and interferon-*γ*.^55^ Uncovering all the molecular networks leading to neuronal death is critical to understanding the responses to inflammation that lead to NDs.

**Figure 7:**
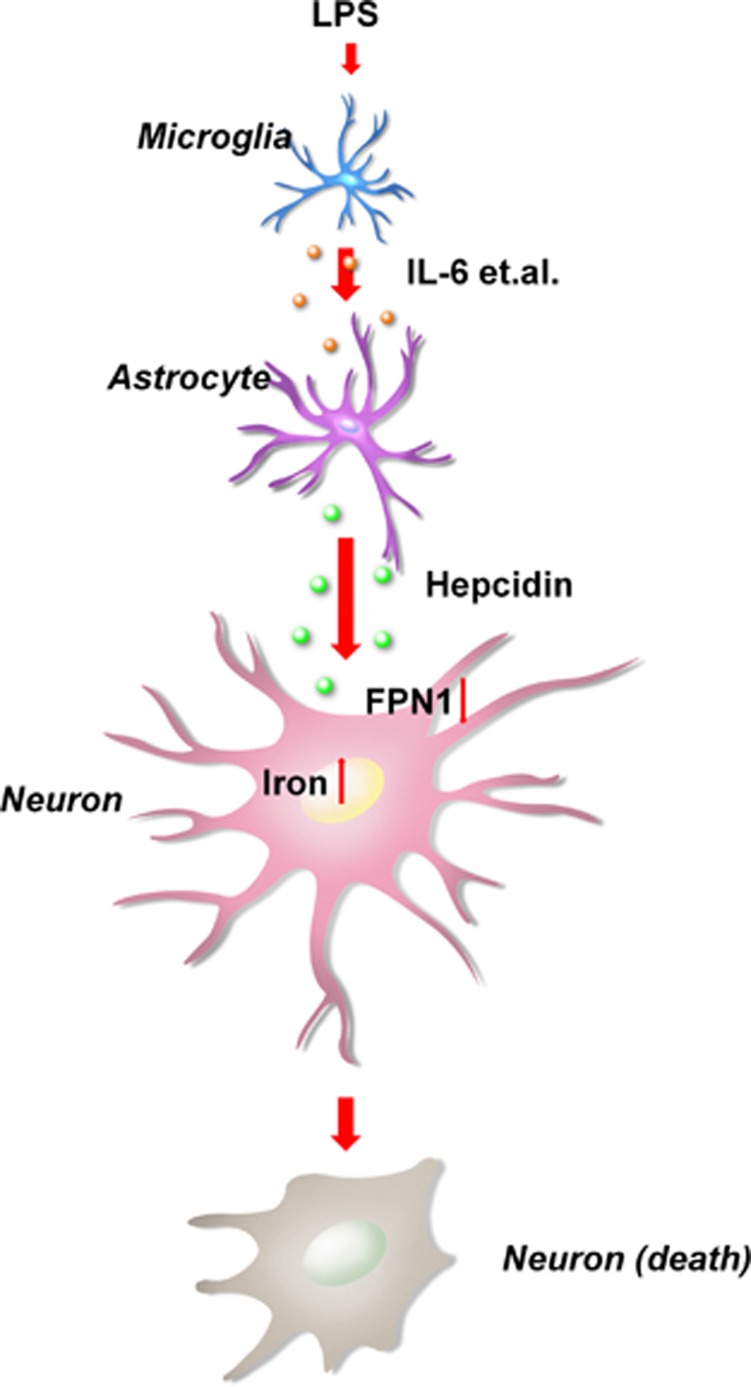
LPS-induced hepcidin expression in astrocytes is regulated by IL-6 from activated microglia. Astrocyte hepcidin is an effector in the pathway of LPS-mediated apoptosis of neurons by decreasing neuronal FPN1 protein levels, which leads to a concomitant increase in neuronal iron levels

In summary, the current study reveals that LPS induces IL-6 expression mainly in microglia. LPS-induced hepcidin expression in astrocytes is regulated by this microglial IL-6. Astrocyte hepcidin then induces the apoptosis of neurons through iron accumulation (Figure 7). Blockage of hepcidin expression in astrocytes resulted in decreased iron overload and apoptosis of neurons in response to LPS. This model linking astrocyte hepcidin and neuroinflammation to iron metabolism dysregulation has important implications in the context of ND pathogenesis.

## Materials and methods

### Animals

All procedures were carried out in accordance with the National Institutes of Health Guide for the Care and Use of Laboratory Animals, and were approved by the Animal Care and Use Committee of Hebei Science and Technical Bureau in PRC. All animals were housed in pairs in stainless steel cages at 21±2 °C and provided free access to food and water. Rooms were humidity controlled room with a 12 h light/dark cycle.

Normal male Balb/C mice, weighing 25 g, were purchased from HEB LAC (Hebei Normal University, Shijiazhuang, China). After mice were allowed to adapt to their living conditions for 3 days, 5 *μ*l LPS (2.5 *μ*g/*μ*l, dissolved in sterile 0.9% saline) or vehicle (sterile 0.9% saline) was infused into the right LCV (0.5 mm posterior, 1.0 mm lateral and 2.0 mm ventral to bregma) as previously described.^13, 50^ The animals were anesthetized (pentobarbital sodium, 40 mg/kg body weight) and then killed for TUNEL and IF, 24 h after LPS injection. The animals were killed for hepcidin mRNA detection 0, 3, 6, 12 or 24 h after LPS injection.

*α*LA (50 mg; Sigma, St. Louis, MO, USA) was weighed and resuspended in 0.35 ml 2 N NaOH. Sterile water (4.5 ml) was added and titrated with approximately 0.05 ml 10 N HCl or until *α*LA slightly precipitated. The pH was adjusted to 7.2–7.4. The solution was filter sterilized through a 0.2 *μ*m low protein binding syringe filter and frozen in aliquots until used. *α*LA (100 mg/kg) was administered intraperitoneally once per day for 2 days. LPS was injected 3 h after the final administration of *α*LA. The animals were anesthetized and then killed for TUNEL and oxidative stress assays 24 h after LPS injection.

Male mice with preferential knockdown of hepcidin in astrocytes were generated by Cyagen Biosciences Inc. (Guang Zhou, China). The vector pRP.ExSi-GFAP-shhepcidin-1 (GFAP promoter – human pre-miR30a flanking sequence – sense–loop–antisense (sense strand: 5′-GCAGACAUUGCGAUACCAATT-3′ antisense strand: 5′-UUGGUAUCGCAAUGUCUGCTT-3′) – human pre-miR30a 3′ flanking sequence – bGH polyA) was microinjected into a fertilized egg. Recombinant embryonic stem cells were injected into FVB blastocysts to produce chimeras, which were then crossed to FVB mice to produce heterozygous mice for preferential knockdown of hepcidin in astrocytes (GFAP-shhepcidin). The right LCVs of GFAP-shhepcidin mice were infused with 5 *μ*l LPS (2.5 *μ*g/*μ*l, dissolved in sterile 0.9% saline) or vehicle (sterile 0.9% saline). The animals were anesthetized and then killed for IF assays 24 h after LPS injection.

The genotype of GFAP-shhepcidin mice was determined using primer-specific PCR. The primer sequences were as follows: Transgene PCR primer forward 5′-AGCTTTATTGCGGTAGTTTATCACA-3′ and reverse 5′-AAAGTAGCCCCTTGAATTCCGA-3′. The PCR products were resolved by agarose gel electrophoresis. Transgenic mice were identified by the presence of a 457 bp-length PCR product.

### Primary cell cultures

#### Rat neocortical neurons

Neurons were prepared from 17-day-old embryonic rat brains, essentially according to the method of Shimojo *et al.*^56^ The fetal rat brains were cleaned by removing the meninges, minced and digested with 0.125% trypsin. Dissociated cells were plated onto poly-l-lysine-coated coverslips at a density of 10^5^ cells per cm^2^ and cultured in minimum essential medium (MEM; Invitrogen, San Diego, CA, USA) containing 10% fetal bovine serum (FBS). Cells were maintained at 37 °C in a humidified atmosphere containing 5% CO_2_ for 8 h and then the medium was switched to Neurobasal medium supplemented with B-27. Subsequently, the medium was changed every 3 days, and cultures were used for experiments following 7 days of culture. With this procedure, at least 95% of the cells in culture were neurons, as evaluated by immunohistochemical staining for NeuN. The cultured medium of neurons was replaced with fresh medium (Dulbecco's modified Eagle's medium (DMEM); the same as used for the C6 cells), without serum 24 h before treatment with C6-conditioned medium.

#### Rat neocortical microglia and astrocytes

Primary cultures of rat microglia and astrocytes were established essentially according to a previously described method.^57, 58^ Briefly, the cortical tissue of newborn rats was dissected in ice-cold D-Hanks' solution. Blood vessels and pia mater were thoroughly removed from the cortex, and the remaining tissue was digested with 0.125% trypsin at 37 °C for 15 min. Mixed cells were seeded in 75 cm^2^ culture flasks at a density of (1.5–2.0) × 10^7^ cells per flask and grown in DMEM containing 10% FBS (Invitrogen), 100 U/ml penicillin and 0.1 mg/ml streptomycin at 37 °C in a humidified atmosphere containing 5% CO_2_. After 24 h any non-adherent cells were removed. Adherent cells were maintained for 9 days with a medium change every 3 days.

After establishment of the mixed glia culture, feeding was stopped at day p10 to allow for significant microglia growth on top of the astrocyte monolayer. The microglia population peaked at 12–14 days in these cultures. To remove any cells adherent to the astrocyte monolayer, microglia-enriched cultures were shaken by rotatory shaking (220 r.p.m. for 50 min at 37 °C). Immediately following agitation, all cells suspended in the culture medium were collected and centrifuged at 300 × *g* for 5 min. The cell pellet was resuspended and diluted with fresh microglia-specific medium bringing the cells to a final concentration of 8 × 10^5^ cells/ml (2 ml/well in a six-well plate). After 30 min, any non-adherent cells were discarded and adherent cells were maintained in fresh microglia-specific medium until assayed. The purity of the microglia cultures was estimated to be over 98% as judged by staining with an antibody against CD11b.

Astrocytes were prepared as follows: mixed glial cultures at day p10 were shaken on a rotatory shaker (220 r.p.m., 24 h) to remove non-astrocyte cells. The astrocyte monolayer was detached and dissociated by trypsin (0.125% trypsin/phosphate-buffered saline (PBS)). The preparation was then filtered using a 100 *μ*m filter screen, and the cell suspension was seeded onto the culture dishes at a density of 0.5 × 10^5^ cells/cm^2^. The purity of the astrocytes was estimated to be 97–98% by staining with antibody against GFAP.

### C6 cell culture and transfection

The rat glial cell C6 line was maintained in DMEM medium containing 10% FBS, 100 U/ml penicillin and 0.1 mg/ml streptomycin at 37 °C with 5% CO_2_.

The sequence of siRNA used to deplete hepcidin was 5′-GTCTCTGTTGCATAACATA-3′. The siRNA was designed by using the OligoEngine software and corresponds to hepcidin nucleotides 148–166 and 190–208. The negative control siRNA is 5′-TTCTCCGAACGTGTCACGT-3′ with no significant homology to any mammalian gene sequence and therefore serves as a non-silencing control. The lentiviral Gv115 vector was provided by the Shanghai Genechem Company (Shanghai, China) and transfected into 293 T cells. The cell supernatants, enriched in viral particles, were collected and concentrated to obtain a high-concentration lentivirus preparation. The final concentration of virus was 5 × 10^8^/ml.

C6 cells were infected using a lentiviral expression system for lentiviral packaging according to the manufacturer's protocols. Generally, cells were plated in six-well plates and cultured to 80% confluence (4 × 10^5^/well). After 24 h the medium was changed and the cells were transfected with lentiviral shhepcidin or with the empty vector. Forty-eight  hours later the media was changed, and the cells were treated with or without IL-6 for 6 h. To assess transfection efficiency, the PCR was used to detect the expression of hepcidin mRNA.

### RNA isolation and quantitative PCR

Primary cultured cells were homogenized in TRIzol reagent (Invitrogen). cDNA was synthesized from 1 *μ*g of the extracted total RNA by using the M-MLV Reverse Transcriptase kit (Takara Biotechnology Co., Dalian, China) according to the manufacturer's protocol.

The level of Hamp1 mRNA was assessed by quantitative PCR as previously described.^13^ PCR amplification was performed with SYBR Green PCR Master Mix (Applied Biosystems, Waltham, MA, USA) via an Applied Biosystems 7500 real-time PCR system with the following cycling parameters: 95 °C for 10 min, followed by 40 cycles of 95 °C for 5 s and then 60 °C for 30 s. *β*-Actin was used as a housekeeping gene control. qPCR primers were designed using Primer Premier 5.0 software and their sequences were as follows: mouse hepcidin forward 5′-TTGCGATACCAATGCAGAAGAG-3′ and reverse 5′-AATTGTTACAGCATTTACAGCAGAAGA-3′ mouse *β*-actin forward 5′-AGGCCCAGAGCAAGAGAGGTA-3′ and reverse 5′-TCTCCATGTCGTCCCAGTTG-3′. Rat IL-6: forward: TCTCCGCAAGAGACTTCCAG; reverse: TTCTGACAGTGCATCATCGCT; rat *β*-actin: forward: 59-GGTCACCCACACTGTGCCCATCTA-39; reverse: 59-GACCGTCAGGCAGCTCATAGCTCT-39; rat Hepcidin: forward: 59-CTATCTCCGGCAACAGACGA-39; reverse: 59-TGAGAGGTCAGGACAAGGCT-39.

Following PCR amplification, a first derivative melting-curve analysis was performed to confirm the specificity of the PCR. The relative fold difference in mRNA between samples was calculated by comparing the threshold cycle (Ct) at which the product initially appeared according to the formula: 2^−^^ΔΔCT^, where ΔCt is the difference between the control group and the treatment group.

### Measurement of iron in neurons

The total iron content of neurons was determined using ICP-MS (Thermo Fisher Scientific, Waltham, MA, USA). Before the experiments, all of the containers were soaked with 15% nitric acid for 24 h, washed with deionized water and then rinsed with ultrapure water before drying. Approximately 1 × 10^6^ cells were added to 1 ml ultrapure nitric acid (69.9–70.0% J.T. Baker, Phillipsburg, NJ, USA) in Teflon digestion tubes, digested in the microwave digestion system for 2 h at 100 °C and then 4 h at 200 °C.^59^ The completely digested samples were diluted to 2 ml with deionized water. Standard curves ranging from 0 to 100 ppb were prepared by diluting an iron standard (1 mg iron/ml) with blanks prepared from homogenization reagents in 0.2% nitric acid. Standards and digested samples were measured in triplicate by ICP-MS.

### Measurement of ROS, MDA and SOD

ROS are generated as a result of the reduction of oxygen during aerobic respiration and by various enzymatic systems within the cell. Levels of ROS were quantified by measuring the fluorescence of DCFH-DA (2′,7′-dichlorofluorescin diacetate), using a commercial kit (Nanjing Jiancheng Bioengineering Institute, Nanjing, China) according to the manufacturer's instructions. Briefly, the cortex or hippocampus of mice was dissected in ice-cold PBS. The tissue was digested with trypsin at 37 °C for 20 min. The preparation was then filtered using a 100 *μ*m filter screen. The cell suspension was collected and centrifuged at 500 × *g* for 10 min. The cell pellet was resuspended and diluted with PBS to a final concentration of 5 × 10^6^ cells/ml. The cells were incubated with 10 *μ*M DCFH-DA for 45 min at 37 °C in the dark. The cells were then washed two times with PBS and then resuspended in PBS. The fluorescence intensity was measured by fluorescence spectrophotometry (Synergy H4; BioTek, Winooski, VT, USA) at an excitation wavelength of 488 nm.

MDA, a marker of lipid peroxidation, was assessed using the thiobarbituric acid (TBA) method^50^ using a kit from the Nanjing Jiancheng Bioengineering Institute according to the manufacturer's instructions. The method is based on the spectrophotometric measurement of the product of the reaction of TBA with MDA. MDA concentrations were then calculated by the absorbance of TBA reactive substances at 532 nm.

SODs, which catalyze the dismutation of superoxide into oxygen and hydrogen peroxide, were determined according to the xanthine oxidase method using a commercial kit (Nanjing Jiancheng Bioengineering Institute) according to the manufacturer's instructions. The xanthine–xanthine oxidase system produces superoxide ions, which can react with 2-(4-iodophenyl)-3-(4-nitrophenol-5-phenlyltetrazolium chloride) to form a red formazan dye, which can be detected by its absorbance at 550 nm^50^

The levels of MDA and the total SOD (T-SOD) activity were determined in each group. The cortex and hippocampus of mice were homogenized in ice-cold saline. The homogenate was centrifuged at 3000 × *g* at 4 °C for 15 min, and the supernatant was used to determine MDA levels and T-SOD activity using a spectrophotometer (Synergy H4; BioTek) at wavelengths of 532 and 550 nm, respectively. Each group contained six mice for the MDA and SOD tests, with each test repeated three times.

### Enzyme-linked immunosorbent assay (ELISA)

The amount of IL-6 release was assayed with ELISA kits (R&D Systems, Minneapolis, MN, USA). IL-6 level in the microglia-conditioned medium collected 24 h after LPS stimulation was determined. Briefly, after LPS treatments, the medium was collected and centrifuged for 20 min at 1000 *g* to remove the pellet. A volume of 10 *μ*l of the supernatant was sampled for measuring IL-6 according to the manufacturer's protocol.

### Western blotting

Protein expression was assessed by western blotting, following polyacrylamide gel electrophoresis as previously described.^50, 60^ The blots were incubated with rabbit anti-rat FPN1 (1 : 5000) (Alpha Diagnostic International, San Antonio, TX, USA), TfR1 (1 : 2000) (Zymed Laboratories, South San Francisco, CA, USA), STAT3 (1 : 2000), P-STAT3 (1 : 2000), rabbit anti-mouse ferritin light chain (1 : 10 000) (FERL14-S; Alpha Diagnostic International) or rabbit anti-mouse ferritin heavy chain (1 : 1000) (ab65080; Abcam, Cambridge, MA, USA) primary antibody overnight at 4 °C. The membrane was washed four times for 15 min with Tris-buffered saline solution containing 0.05% Tween 20 and then incubated with anti-rabbit (RPN4301; Amersham, Buckinghamshire, UK) or anti-mouse IgG secondary antibody (RPN4201; Amersham) conjugated to horseradish peroxide (1 : 5000) for 90 min at room temperature. Peroxidase activity was detected with the SuperSignal WestPico chemiluminescent substrate (Pierce Biotechnology, Waltham, MA, USA) and visualized and digitized with ImageQuant (Fujifilm LAS-4000, Tokyo, Japan). Optical densities of bands were analyzed using Multi Gauge V3.1 (Fujifilm, Tokyo, Japan). All experiments were carried out at least three times. The relative band intensities of the proteins are presented in comparison with that of *β*-actin (A5060; Sigma-Aldrich, St. Louis, MO, USA).

### Immunofluorescence

Under anesthesia with Nembutal, the animals were perfused with 0.9% saline followed by 4% paraformaldehyde in 0.1 M phosphate buffer (PB, pH 7.2–7.4). The brains were removed, postfixed for 1.5–4 h in 4% paraformaldehyde in PB and then stored overnight in 30% sucrose. Serial coronal sections were cut to a thickness of 30 *μ*m on a freezing microtome and mounted onto a slide covered with 3-aminopropyl-triethoxysilane (APES; Beijing ZhongShan Biotechnology, Beijing, China). The slices were washed with 0.01 M PBAS (PBS, pH 7.2–7.4) before incubation in 3% H_2_O_2_ for 10 min to quench endogenous peroxidase activity. Antigen retrieval was performed in a microwave oven for 10 min in 10 mM citrate buffer (pH 6.0). After blocking for 1 h with normal goat serum prepared in 0.01 M PBS, the slices were incubated overnight at 4 °C with mouse anti-GFAP monoclonal antibody (1:400) (MAB360; Millipore, Temecula, CA, USA), mouse anti-CD11b monoclonal antibody (1:400) (ab1211; Abcam), anti-NeuN monoclonal antibody (1:100) (MAB377; Millipore), rabbit anti-FPN1 antibody (1:400) (MTP11-A; Alpha Diagnostic International), rabbit anti-caspase-3 (1:300) (9662 and 9661; Cell Signaling Technology, Danvers, MA, USA), rabbit anti-mouse ferritin light chain (1:10 000) (FERL14-S; Alpha Diagnostic International) or rabbit anti-mouse ferritin heavy chain (1 : 200) (ab65080; Abcam). The slides were then washed three times with 0.01 M PBS for 5 min. The following secondary antibodies were used in a 60 min incubation at 37 °C: rhodamine-conjugated goat anti-rabbit IgG (1:200) (AP132R; Millipore Corporation, Temecula, CA, USA), FITC-conjugated goat anti-mouse IgG (1:200) (AP124F; Millipore Corporation). Finally, after washing and mounting, the sections were photographed with a Zeiss LSM710 microscope.

### Double IF staining – TUNEL and NeuN

TUNEL staining was conducted using a commercial kit (*In Situ* Cell Death Detection Kit; Roche, Penzberg, Germany), following the manufacturer's instructions. In brief, tissue slides were pretreated with 20 *μ*g/ml proteinase K for 5 min and then incubated with the reaction mixture containing terminal deoxynucleotidyl transferase (TdT) and fluorescein-conjugated deoxyuridine triphosphate (dUTP) for 1 h at 37 °C. The sections were washed with PBS, following which serum was added and the samples incubated at 37 °C for 30 min. Subsequently, these sections were incubated at 4 °C overnight after the addition of primary antibody (anti-NeuN monoclonal antibody, 1:100, MAB377; Millipore) and then incubated at room temperature for 2 h after the addition of secondary antibody (goat anti-mouse IgG H&L (DyLights 549), 1 : 200, ab96885; Abcam). Finally, after washing and mounting, the sections were analyzed with a Zeiss LSM710 microscope.

### MTT assay

The MTT assay was used to evaluate cell proliferation. Primary neurons (1 × 10^5^ cells/well) were cultured in DMEM medium supplemented with B-27 on 96-well plates overnight, then exposed to the various treatments for 24 h. The neurons were then exposed to 20 *μ*l MTT (0.5 mg/ml; Sigma) for 3–4 h and the resulting formazan was dissolved in 150 *μ*l DMSO. The absorbance at 570 nm (A570) was recorded using a microplate reader (Berkeley; Bio-Rad, Hercules, CA, USA). The percentage of viable cells was calculated using the following formula: survival (%)=A570 (sample)/A570 (control) × 100%.

### Statistical analysis

All the data are presented as the mean±S.D. The statistical analyses of group differences were assessed by one-way ANOVA followed by either the Dunnet test or Student–Newman–Keul's test (as a *post hoc* test). Differences were considered significant when *P*<0.05. All the tests were performed with SPSS 21.0.
