# Supplementary material for: Astrocyte hepcidin is a key factor in LPS-induced neuronal apoptosis
Source: Cell Death Dis. 2017 Mar 16;8(3):e2676–. doi: 10.1038/cddis.2017.93 (PMC5386583; doi:10.1038/cddis.2017.93)
Supplement: Supplementary Information [file cddis201793x1.docx]

**Astrocyte Hepcidin is a Key Factor in LPS-induced Neuronal Apoptosis**

Lin-Hao You^1,#^, Cai-Zhen Yan^1,2#^, Bing-Jie Zheng^1^, Yun-Zhe Ci^1^, Shi-Yang Chang^1^, Peng Yu^1^, Guo-fen Gao^1^, Hai-Yan Li^1^, Tian-yu Dong^1^, Yan-Zhong Chang^1^*

^1^ Laboratory of molecular iron metabolism, The Key Laboratory of Animal Physiology, Biochemistry and Molecular Biology of Hebei Province, College of Life Science, Hebei Normal University, Shijiazhuang, China

^2^ School of Basic Medical Sciences, Hebei Medical University, Shijiazhuang, China

^#^ Lin-Hao You and Cai-Zhen Yan contributed equally to this work.

* Corresponding author.

Yan-Zhong Chang, PhD, Professor,

Laboratory of Molecular Iron Metabolism,

College of Life Science,

Hebei Normal University,

Shijiazhuang, Hebei Province, 050024,

China.

E-mail: frankyzchang@yahoo.com.hk,

Tel: 86-311-80786311,

Fax: 86-311-80786311.

**Supplementary Information**


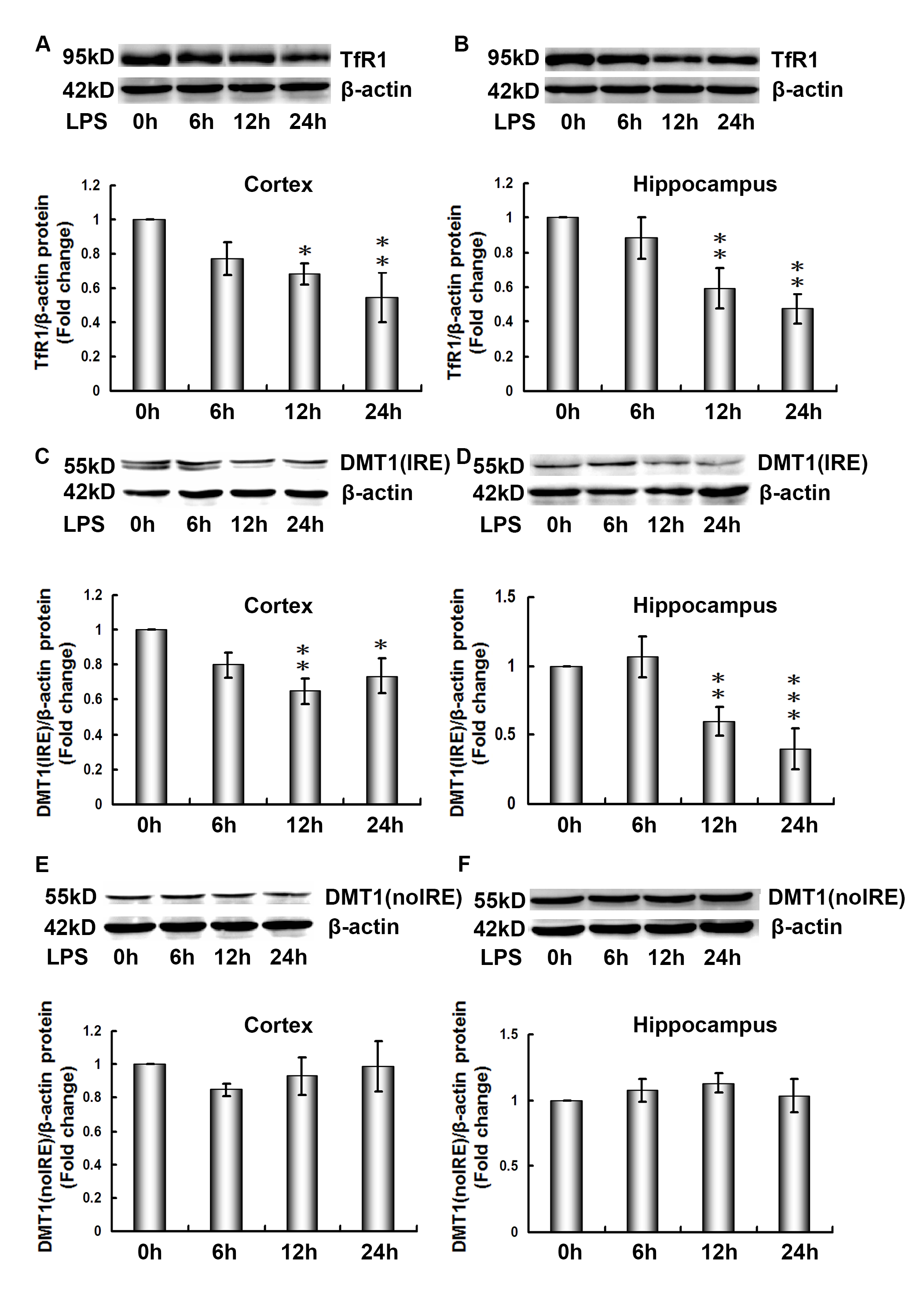


**Supplementary Figure S1** The expression of iron uptake related proteins in the cortex and hippocampus following LPS injection. The TfR1 (A-B), DMT1+IRE (C-D) and DMT1-IRE (E-F) protein levels in these regions of the brain at 0, 3, 6, 12 and 24 h after LPS injection. Expression levels were normalized to β-actin and presented as the mean ± SD. **p<*0.05, ***p<*0.01, and ****p<*0.001 vs. 0 h.


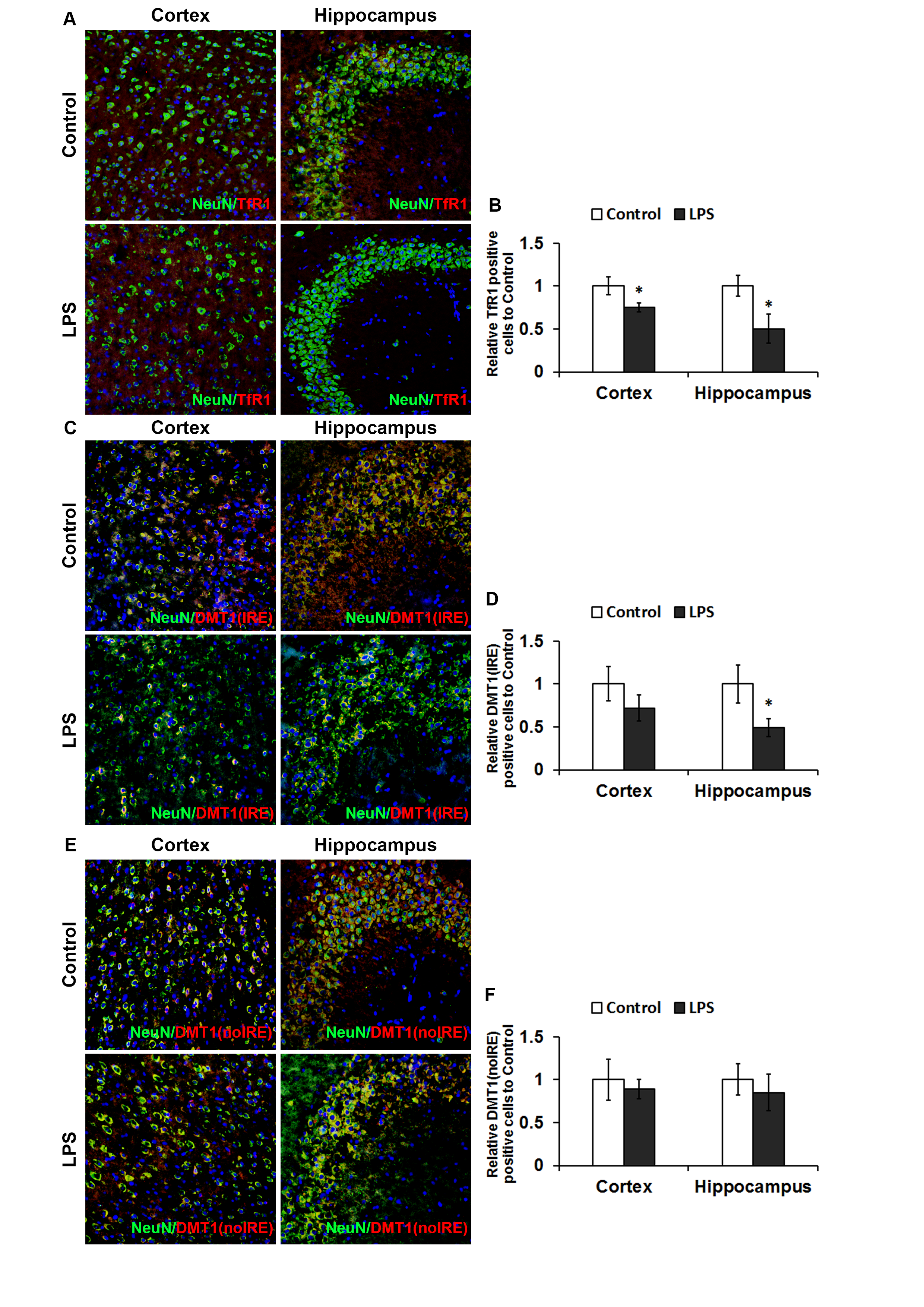


**Supplementary Figure S2** The expression of iron uptake related proteins in neurons in the cortex and hippocampus following LPS injection. Double immunoﬂuorescence labeling of TfR1 (A-B), DMT1 (IRE) (C-D) or DMT1 (noIRE) (E-F) and NeuN (staining for neurons) was carried out in cortical and hippocampal sections. Scale bar=100 μm. Values are presented as the mean ± SD. **p*<0.05 vs. control group.


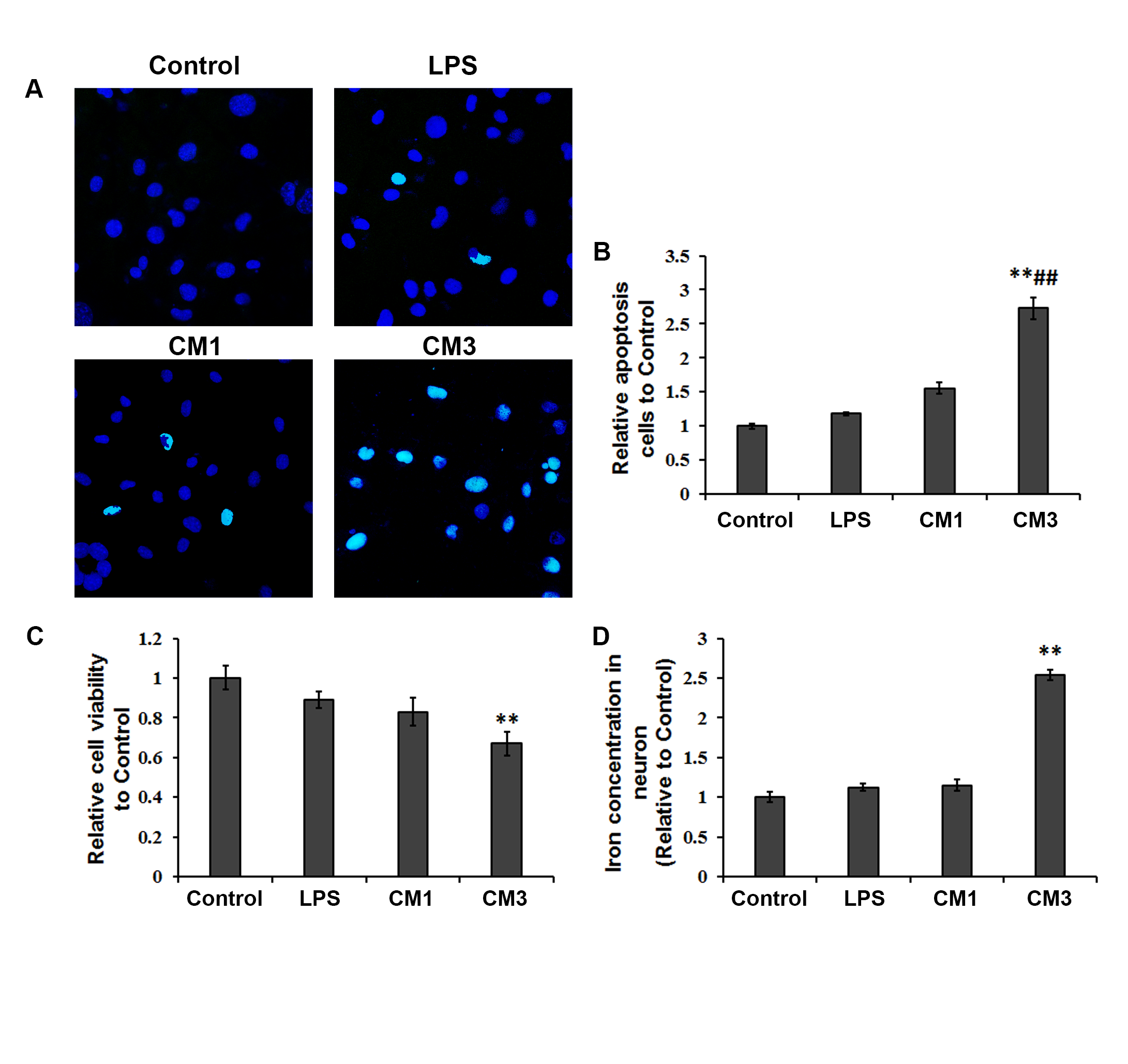


**Supplementary Figure S3** Activated glial cells contributes to the death of neurons following LPS injection. (A) Primary neurons were stained with DAPI and TUNEL, then examined by ﬂuorescence microscopy following LPS, CM1 and CM3 treatment for 24 h. (B) Statistical analysis of apoptosis of primary neurons using TUNEL staining. Data were expressed as mean ± SD. ***p*<0.01 vs. control group; ##*p*<0.01 vs. LPS treatment group. (C) Statistical analysis of cell viability detected by MTT after LPS, CM1 and CM3 treatment for 24 h. Data were expressed as mean±SD. ***p*<0.01 vs. control group. (D) Total iron of primary neurons was measured by ICP-MS after LPS, CM1 and CM3 treatment for 24 h. All data represent the mean value of five separate experiments (three replicates per experiment).Data were expressed as mean±SD. ***p*<0.01 vs. control group. CM1: microglia medium collected 24 h after LPS stimulation. CM3: astrocyte medium collected 24 h after CM1 treatment.


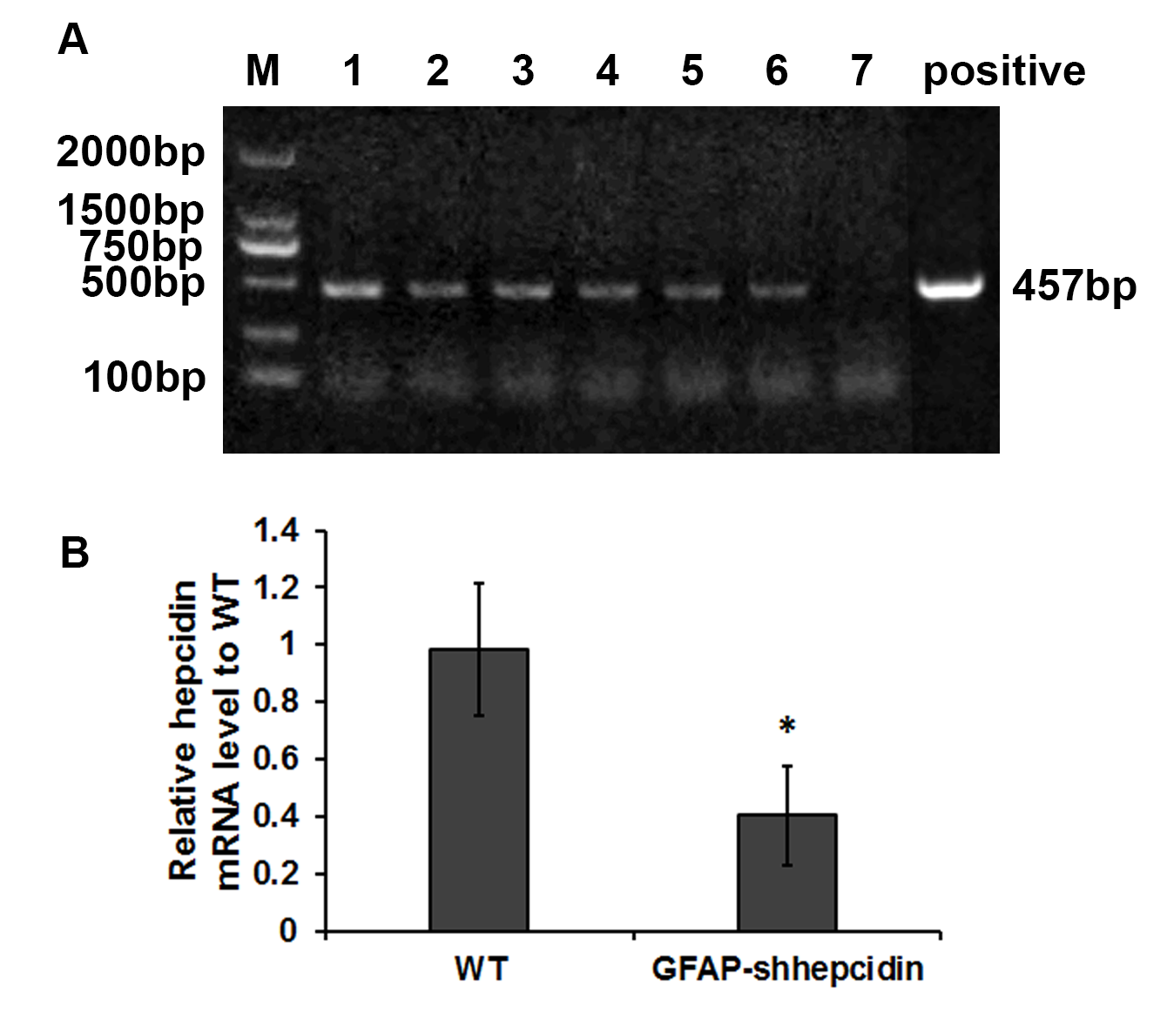


**Supplementary Figure S4** Identification of transgenic mice with preferential knockdown of hepcidin in astrocytes. (A) The genotype of mice used in this study was identified by primer-specific PCR. The mice, which possessed the 457bp-length PCR products, were transgenic mice such as No. 1 to 6. The mice without 457bp-length PCR product were wild-type mice such as No. 7. M: marker; positive control: an amount of transgene injection vector. (B) Hepcidin expression was evaluated by PCR in primary cultures of astrocytes extracted from the GFAP-shhepcidin mice. Hepcidin mRNA expression was detected by real-time PCR in WT or GFAP-shhepcidin mice. Expression levels were normalized to β-actin and presented as the mean ± SD. **p*<0.05 vs. WT group.
